# Prediction of midline shift after media ischemia using computed tomography perfusion

**DOI:** 10.1186/s12880-022-00762-0

**Published:** 2022-03-12

**Authors:** Sebastian Johannes Müller, Eya Khadhraoui, Marielle Sophie Ernst, Christian Heiner Riedel

**Affiliations:** Department of Neuroradiology, University Hospital Göttingen, Georg-August-University, Göttingen, Germany

**Keywords:** Media ischemia, Perfusion, DHC, Hemicraniectomy, KM index

## Abstract

**Background:**

Decision-making about the indication for decompressive hemicraniectomy in cases with malignant infarction in the territory of the middle cerebral artery (MCA) is still a matter of debate. Some scores have been introduced and tested, most of them are midline-shift dependent. We introduce the Kinematics of malignant MCA infarction (KM) index, which can be calculated based on an initial computed tomography perfusion scan and the chosen therapy (lysis/thrombectomy/conservative) in order to estimate the maximum midline-shift in the subsequent 6 days.

**Methods:**

We retrospectively analyzed patients with middle cerebral artery infarction who had a non-enhanced computed tomography (CT) scan, CT angiography and a CT perfusion scan in the acute setting and who presented in our emergency room between 2015 and 2019. 186 patients were included. Midline shift was measured on follow-up imaging between days 0 and 6 after stroke. We evaluated Pearson’s correlation between the KM index and the amount of midline shift.

**Results:**

The mean KM index of all patients was 1.01 ± 0.09 (decompressive hemicraniectomy subgroup 1.13 ± 0.13; midline shift subgroup 1.18 ± 0.13). The correlation coefficient between the KM index and substantial midline-shift was 0.61, *p* < 0.01 and between KM index and decompressive hemicraniectomy or death 0.47; *p* < 0.05. KM index > 1.02 shows a sensitivity of 92% (22/24) and a specificity of 78% (126/162) for detecting midline shifts. The area under curve of the receiver operator characteristics was 91% for midline shifts and 86% for the occurrence of decompressive hemicraniectomy or death.

**Conclusion:**

In this retrospective study, KM index shows a strong correlation with significant midline-shift. The KM index can be used for risk classification regarding herniation and the need of decompressive hemicraniectomy.

## Background

Decision-making for decompressive hemicraniectomy (DHC) after media ischemia is very complex. Decompressive surgery for the treatment of malignant infarction of the middle cerebral artery (DESTINY) and DESTINY2 studies showed a survival benefit for a distinct patient population after DHC [1, 2]. Specifically, accurate indication and time of surgery are important. In order to identify patients, who need DHC, several scores have been introduced and tested. Among these, the enhanced detection of edema in malignant anterior circulation stroke (EDEMA) score [3], which is strongly midline shift-dependent (thresholds for DHC > 6 mm and > 9 mm), is often used. If the patient neurologically worsens, they need to be intubated, a non-enhanced computed tomography (NECT) scan has to be performed and the patient has to be transferred to the operating room. In some cases, a living will of the patient is unavailable. All these time delays can lead to herniation, further neurological worsening and death. A tool offering earlier prediction of critical swelling is urgently needed. We introduce a new index and score, the Kinematics of Media ischemia (KM) index, which can be calculated based on the initial computed tomography perfusion (CTP) scan and which makes use of the chosen therapy in reference cases (lysis/thrombectomy/conservative) for estimating the maximum midline shift (MLS) in the subsequent 6 days. The KM index serves as an indicator for timely decision making regarding DHC.

## Materials and methods

We retrospectively analyzed patients with middle cerebral artery infarction who had a non-enhanced computed tomography (NECT) scan, Computed tomography angiography (CTA) and CTP scan in the acute setting. The considered time interval was between 01.01.2015 and 30.06.2019.

### Inclusion and exclusion criteria

Patients with acute unilateral middle cerebral artery occlusion who had an initial CTP scan and at least one imaging study in the following 6 days were included. Patients were excluded if CTP scans were not sufficiently diagnostic due to technical issues. Patients with large intracranial hemorrhage and/or serious iatrogenic complications (intracranial bleedings > 20 ml) were also excluded. Full criteria are listed in Table 1.

**Table 1:** Inclusion and exclusion criteria

Inclusion criteria	Exclusion criteria
Acute unilateral middle cerebral artery occlusion	Large intracranial hemorrhage
Initial CTP scan	Serious iatrogenic complications (intracranial bleedings > 20 ml)
NECT scan (or MRI) control in the first 6 days after infarction	CTP scans with technical issues or no medium

The table demonstrates the inclusion and exclusion criteria for the patients

### Factors influencing MLS

Three CTP-dependent factors were determined by standard post-processing software tools: (1) volumes of infarction (I), (2) volume of penumbra (P) and (3) brain parenchyma (B).

Additionally, the following clinical criteria were examined: (4) prehospital delay, (5) lysis and (6) result of thrombectomy.

We analyzed the correlation of MLS with these single factors as well as with their linear combinations.

### Factor dependencies

Since the prehospital delay (the time of symptom onset until the time of hospital arrival) is influencing the decision for lysis and thrombectomy, it seems dependent and may not be useful in our concept.

### Volumes of penumbra, infarct and brain parenchyma

We only use ratios and no absolute volumes to reflect potentially protective brain atrophy. As shown in Eq. (1), B in percentage is calculated:

1 $$B=1-\frac{\left(vs+vv\right)}{iv}$$

iv = intracranial volume, vs = volume of subarachnoid spaces; vv = volume of ventricle.

As reflected in Eq. (2), the size of the potential infarction and penumbra in percentage are determined by:

2 $$I=\frac{vc}{iv};\quad P=\frac{vp}{iv}$$

vc = volume of core, vp = volume of penumbra.

### Parameters m and n

Successful thrombectomies are reducing the volume of infarction in the penumbra and thus the amount of cerebral edema. Additional weighting factors m and n are added to the KM index to reflect this impact. The degree of swelling of brain tissue after infarction is represented by the parameter m. The probability of the infarction of the penumbra is reflected by the parameter n. Both variables depend on the treatment results.

### Thrombectomy and intravenous thrombolysis

Thrombectomy results were documented using the mTICI-Score [4]. In case of a timely and successful recanalization of an artery, the penumbra can be saved from further infarction. We multiplied a weighting factor n for the result of the thrombectomy with P, the ratio of the volume of the penumbra/volume of intracranial space. If no thrombectomy was performed, n was set to 1.0. The weighting factor n after thrombectomy is shown in Table 2. An additional factor λ was analyzed for the influence of intravenous thrombolysis with recombinant tissue plasminogen activator (rtPA).

**Table 2:** Weighting factor n

mTICI-Score	Weighting factor n without rtPA	Weighting factor n with rtPA (e.g. λ = 0.7)
0	1.0	0.7
1	0.9	0.63
2a	0.8	0.56
2b	0.7	0.49
2c	0.6	0.42
3	0.5	0.35

The weighting factor n based on lysis and thrombectomy result is shown

We decided to use a linear model despite the fact that outcomes of thrombectomies classified by mTICI are not quantitatively separated at intervals.

### The KM index

If the volume of swollen tissue is higher than the volume of the free intracranial space, an MLS is expected. In order to incorporate this simple rule, we add the following 3 summands and assume that MLS is implausible, if KM index is smaller than 1. The basic formula is shown in Eq. (3).

3 $$KM{\text{-}}Index=m*I +n*P+B$$

For healthy brains the following applies: $n=m=0$ and $KM{\text{-}}Index < 1$. For easier computation, we set the parameter $0\le n\le 1$. We assume a swelling potential of an infarcted volume with maximum of 400% of the original area. We tested from $m=0.1*n$ up to $m=5.0*n$. We approximated the optimal parameters by choosing the highest correlation of KM index and MLS in our retrospective cohort. A threshold analysis of the $KM{\text{-}}Index> x$, for assuming the occurrence of MLS and DHC was performed.

### “Time is brain”

Time as a factor is implicitly included in the KM index, because it depends on the timely performance and results of thrombectomy and intravenous thrombolysis. Whether this inclusion is sufficient seems unclear. So, we added an additional factor t, as demonstrated in Table 3, for the time between symptom onset and thrombectomy (time to groin puncture), to predict the time dependence of the MLS. The additional index was tested as well, as shown in Eq. (4).

**Table 3:** Time factor t

Time between onset and thrombectomy	“t”
0–4.5 h	3
4.5–8 h	2
> 8 h	1

The additional time factor t was tested as well

4 $$KMT{\text{-}}Index={m}^{t}*I +{n}^{t}*P+B$$

### Measurement of midline shifts

The measurement of midline shifts was performed both on transverse and coronal slices (each reconstructed with 4 mm slice thickness) [5]. The maximum of both values was used for statistical analysis. The location of maximum MLS was not considered.

### Other clinical factors

Pretreatment National Institutes of Health Stroke Scale (NIHSS), age of patient, diabetes as co-disease and a novel oral anticoagulants (NOAC) therapy were also analyzed. For the sake of simplicity and because of the dependence on the lysis decision, we have not included these factors in the KM index.

### Control group

Since we optimized the index parameters m and n based on the existing cohort, we decided to re-test the KM index on a sample of patients who presented between 01.07.2019 and 31.10.2019.

### Perfusion-settings

The standardized stroke imaging was performed using a 128-slice multidetector CT scanner (Siemens Definition AS+; Siemens Healthcare Sector, Forchheim, Germany). It encompassed an NECT, followed by CTP with 9 cm coverage and a single phase CTA of the head and neck. CTP contained 930 images (31 × 30 consecutive spiral scans of the brain, 45 s acquisition time, tube tension was 80 keV, effective dose of approx. 5 mSv). An injection of 36 ml of contrast agent (Imeron) was continuously performed with a flow rate of 4.5 ml/s through the antecubital vein and finished with a chaser bolus of 30 ml of physiologic salt solution. Reconstruction of CTP data was done with a slice thickness of 5 mm every 3 mm. Further data analysis was done using Syngo.via™ (Siemens^®^) with automatic motion correction and standardized parameters. Patients diagnosed with thrombotic large vessel occlusion and mismatches were transferred to the angiography suite for mechanical thrombectomy.

### Volume-ratio calculation

Volume analysis was performed using the CT Neuro Perfusion module of Syngo.via™ (Siemens Healthcare GmbH, Henkestrasse 127, 91052 Erlangen, Germany). A neuroradiology fellow (> 4 years’ experience in CT diagnostics, blinded to clinical information) evaluated the cases and checked the automated segmentation for errors.

**Fig. 1:**
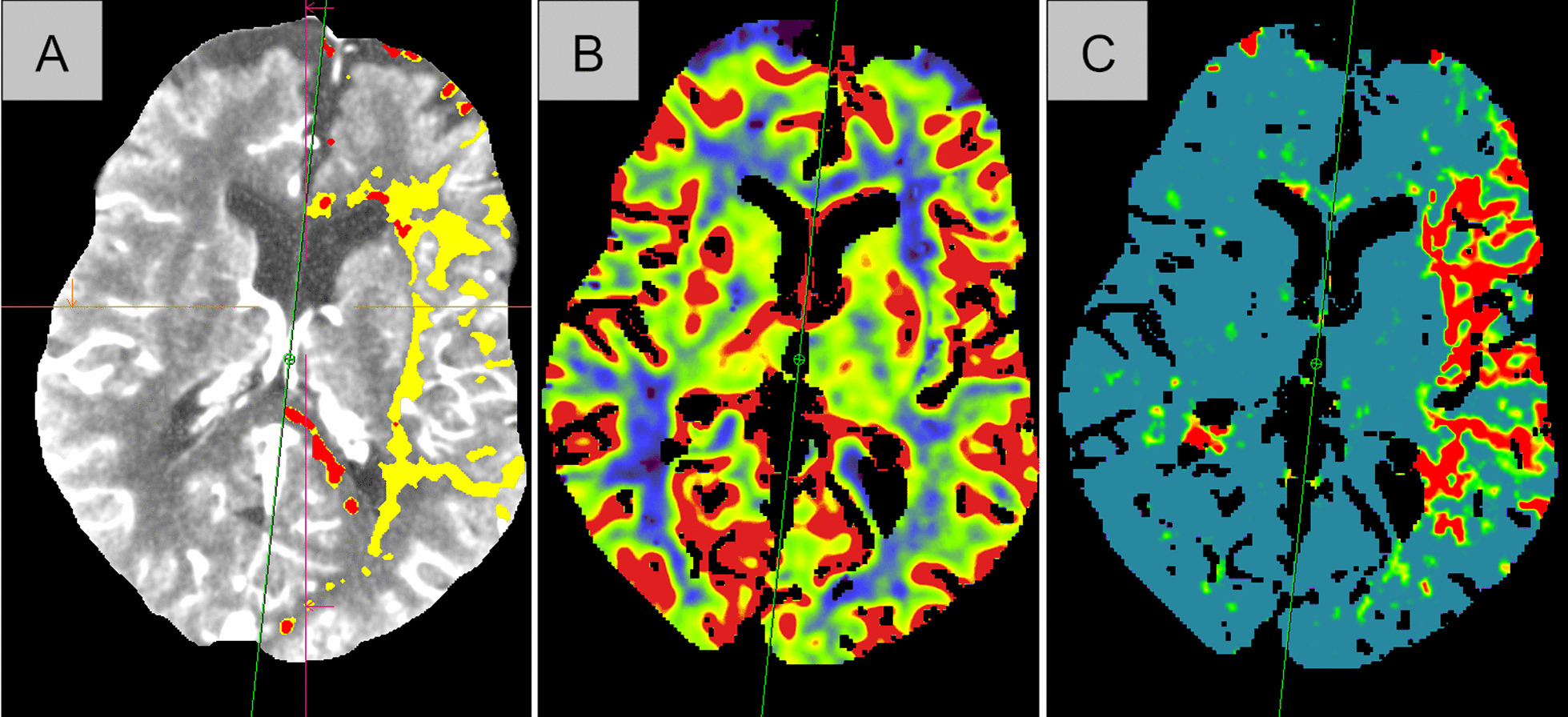
Voxel volume analysis. **A** MIP-partial intracranial volume; **B** CBV; **C** MTT-partial brain volume. *MIP* maximum intensity projection, *CBV* cerebral blood volume, *MTT* mean transit time. In **B**, the blood volume is represented by the colors red–yellow–blue (descending). In **C**, areas with a normal transit time are marked blue, an area with a delay (penumbra) is marked from green to yellow, while an acute infarction is shown in red

**Fig. 2:**
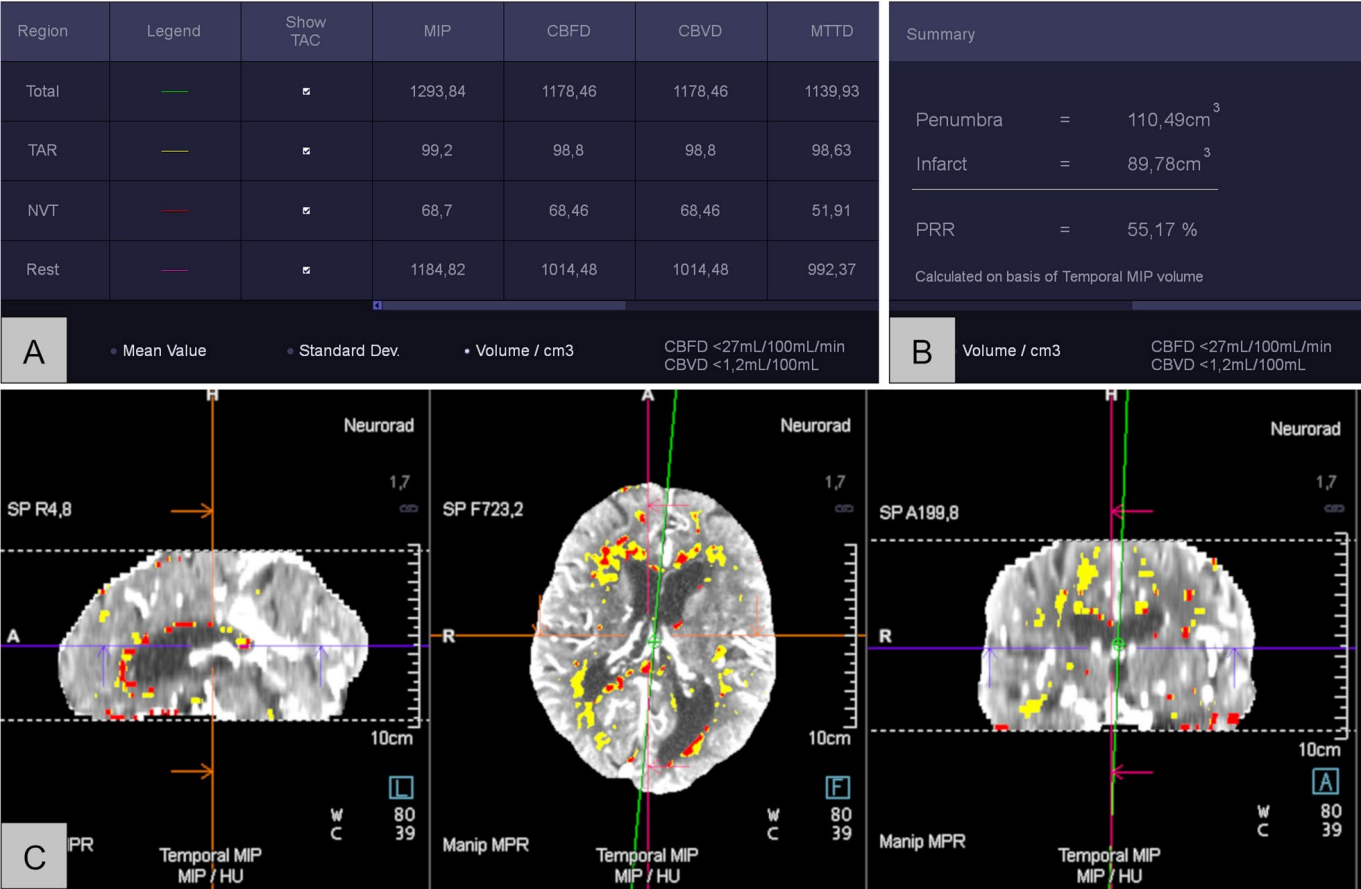
Screenshots of the Syngo.via Software. **A** Collecting the volumes of MIP/CBF/CBV and MTT, **B** penumbra and Infarct volume acquisition, **C** topogram of CTP. *MIP* maximum intensity projection, *CBF* cerebral blood flow, *CBV* cerebral blood volume, *MTT* mean transit time, *CTP* computed tomography perfusion, *TAR* tissue at risk, *NVT* non-viable tissue, *TAC* time attenuation curve, *PRR* penumbra relative ratio, *HU* Hounsfield unit, *MPR* multiplane reconstruction

As volume rendering of infarct, penumbra and brain is not pragmatically applicable yet, we decided to use the CTP data analyzed with Syngo.via™ Siemens^®^. An easy calculation of volume ratios was done using Syngo.via’s perfusion-voxel-data. As shown in Figs. 1 and 2 we used the automated assessment of partial intracranial volume (MIP), cerebral brain volume (CBV) and mean transit time (MTT) voxel volume analysis and the automatically derived infarct core and penumbra estimation. Atrophy plays an important protective role in herniation after brain ischemia and is included in this ratio. The brain volume results from $OS = 1-CFS$. The ratio $CFS=MTT/MIP$ was used as an estimation of the cerebral fluid space (CFS).

### Statistical analysis

The program Statistica, version 13 (TIBCO Software Inc., Palo Alto, CALIFORNIA, USA) was used. *p* values below 0.05 were defined as statistically significant. Pearson’s correlation was calculated for correlation testing between MLS and KM index, DHC; infarct volume and further variables. A Receiver Operating characteristics (ROC)- and Area Under the Curve (AUC)-Test combined with Mann–Whitney-U-Test was used to derive optimal thresholds for the sensitivity/specificity of the KM index. Values are given in standardized form: mean ± standard deviation.

## Results

### Patients

We analyzed 261 patients with middle cerebral artery infarction and initial CTP. 75 patients were excluded due to bad quality of CT perfusion (n = 40), complications during interventions (n = 6) or missing follow-up NECT scans (n = 29). 186 patients were included.

The mean age was 73.7 ± 17.9 years (mean ± standard deviation). 109 (58.6%) were females. In 51.1% (95 cases) the right middle cerebral artery (MCA) territory was affected. 24 patients suffered from a wake-up stroke. In 27 cases the time of symptoms onset was unclear. The distribution of large vessel occlusions and the count of resulting MLS are displayed in Table 4.

**Table 4:** Occluded vessels

Artery	Cases	No. of MLS’s
CCA	4	1 (25%)
ICA	28	8 (29%)
Carotid T	13	6 (46%)
M1 of MCA	136	15 (11%)
M2 of MCA	28	1 (4%)
M3 of MCA	3	0 (0%)
Combined	26	8 (31%)

The distribution of vessel occlusions found in patients included in the KM index study

### Other clinical data

Mean NIHSS at time of CT was 9.5 ± 2.1. Forty-five patients (24%) suffered from type 2 diabetes: 114 patients (61%) were not anticoagulated before admission.

For the subgroup of patients with a midline-shift pre-treatment NIHSS was 11.0 ± 2.4, the diabetes rate 20.8% and 62.5% of patients were not anticoagulated.

### CT and CTP data

The mean volumes computed with Syngo.via were v(P) = 87.77 ± 37.15 cm^3^, v(I) = 53.81 ± 49.71 cm^3^, v(MIP) = 1086.26 ± 105.59 cm^3^, v(CBF) = 987.08 ± 99.34 cm^3^ and v(MTT) = 950.27 ± 105.69 cm^3^. The mean occupied space was 0.908 ± 0.109. A correlation with the age of patients was significant (Correlation coefficient r = − 0.197, significance α < 0.05).

### MLS and DHC

Follow-up NECT Scans between day 1 and day 6 after stroke onset were analyzed. 297 follow-up CT scans were evaluated (in average 1.6 per patient). The mean time interval between initial scan and first follow-up CT was 1.5 ± 1.3 days (median 1, range 1–6). All MLS’s were documented and the maximum MLS was evaluated. The mean MLS was 0.83 ± 2.58 mm. Only in 24 of 186 cases an MLS was observed. In 14 cases the patients underwent a DHC. The final infarct volume was not calculated.

### Thrombectomies

132 of 186 patients underwent mechanical thrombectomy. Thrombectomy results were as follows: mTICI 0 in 12 cases, mTICI 1 in 2 cases, mTICI 2a in 10 cases, mTICI 2b in 33 cases, mTICI 2c in 9 cases and mTICI 3 in 66 cases.

### Lysis

In 94 patients (50.53%), an intravenous thrombolysis with rtPA was performed.

### Single correlations of MLS

The correlations of MLS with the single factors are listed in Table 5. Penumbra, MTT/MIP and lysis did not significantly correlate. The factors “n” and “I” were the best predictive markers. DHC shows the best correlations as a dependent control marker.

**Table 5:** Univariate correlation analysis for predictors of MLS

Factor	Correlation coefficient R (with MLS)	Correlation function and gradient M	*p* value
P	0.083	0.08 + 0.0011*MLS	NS
I	0.327	0.045 + 0.0055*MLS	< 0.05
MTT/MIP	0.045	0.91 + 0.0019*MLS	NS
Lysis	− 0.128	0.535–0.0244*MLS	NS
Factor n without Lysis	0.325	0.712 + 0.027*MLS	< 0.05
Factor n with Lysis	0.340	0.665 + 0.028*MLS	< 0.05
Factor t	− 0.172	2.37–0.058*MLS	< 0.05
*DHC*	0.625	0.022 + 0.064*MLS	< 0.05

Single factor analysis of measured and calculated values with measured midline shifts

*NS not significant (*p* > 0.05)

### Indexes and optimizing of parameters

We assumed a linear negative correlation between the result of thrombectomy and MLS. Hence we simply set n = 0.5 for mTICI 3, n = 1 for mTICI 0. Finding the right weighting of lysis was achieved by using iterative algorithms to maximize the correlation of KM index and MLS. The approximated optimal value was λ = 0.70. Without using OS in the formula, all correlation coefficients decreased by approx. 0.04, so we still assume a measurable, but low influence of atrophy. Using it as a factor decreased the correlation as well.

### KM index of Syngo.via

Since our approximation procedures showed $m=2.8*n$ being the optimal correlation value for our retrospective patient cohort using Syngo.via, the KM index is shown in Eq. (5):

5 $$KM{\text{-}}Index \left(Syngo.via\right)=\frac{\begin{array}{c}n*(2.8*v\left(I\right)+ v\left(P\right)) + v(MTT)\end{array}}{v(MIP)}$$

### “Time is brain”

Trying to use an additional relationship with time, we set $\mathrm{n}={\mathrm{n}}^{\mathrm{t}}$ and repeated the optimizing process finding λ(t) = 0.87 to be the best value. The correlation with MLS showed equal results compared to the KM index for $m=\sqrt[t]{2.8}*n$, see Eqs. (6) and (7):

6 $$KMT{\text{-}}Index =\frac{{m}^{t}*vI + {n}^{t}*vP + vMTT}{vMIP}$$

7 $$KMT{\text{-}}Index \left(Syngo.via\right)=\frac{{2.8*n}^{t}*( vI+vP) + vMTT}{vMIP}$$

### Correlation of KM index with MLS

**Fig. 3:**
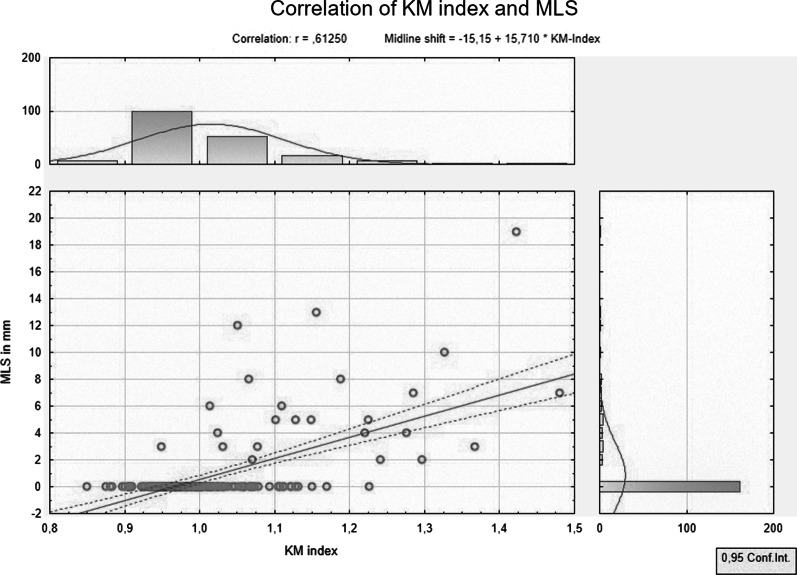
Correlation of KM index and detected midline shift. The graph demonstrates the correlation of KM index and midline shift and the rough distribution of the calculated and measured values. Line—correlation, dotted lines—regression bands (confidence)

The mean KM index for all patients was found to be 1.014 ± 0.094. The KM index of the DHC subgroup was 1.129 ± 0.128 and for the MLS group it was 1.177 ± 0.131. The correlation coefficient between the KM index and maximal MLS was 0.6125; significance α < 0.05, as demonstrated in Fig. 3.

### Finding the right cut-off value for MLS and DHC

**Fig. 4:**
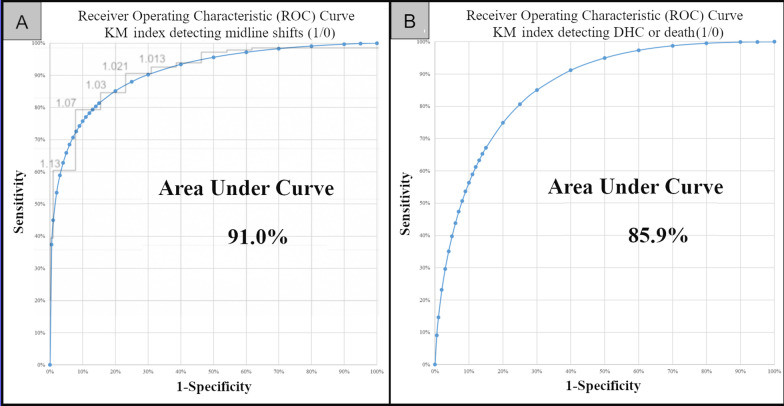
Receiver operating characteristics of the KM index. **A** RoC Curve KM index and midline shift; **B** RoC Curve KM index and DHC or death. *RoC* receiver operating characteristics, *DHC* decompressive hemicraniectomy

In only one case with KM index < 1.01 a MLS was detected. The optimal KM > 1.021 shows a sensitivity of 91.6% (22/24) and a specificity of 77.8% (126/162) for detecting MLS’s. Receiver operating characteristics are shown in Fig. 4.

The sensitivity of a KM index above 1.13 for a significant MLS was 76% (13/17). Seven of these 17 patients underwent DHC, three underwent best supportive care and died. Seven survived with conservative therapy.

### Control group

Since we calculated and optimized our formula on a set of 186 patients, the results on this selected patient cohort were excellent, as expected. For verification, we calculated the KM index on a control group of 24 patients. In these small cohort (only 7 patients with MLS), we observed similar results for the correlation (correlation coefficient 0.58, significance α < 0.05) with MLS.

### KM score

**Fig. 5:**
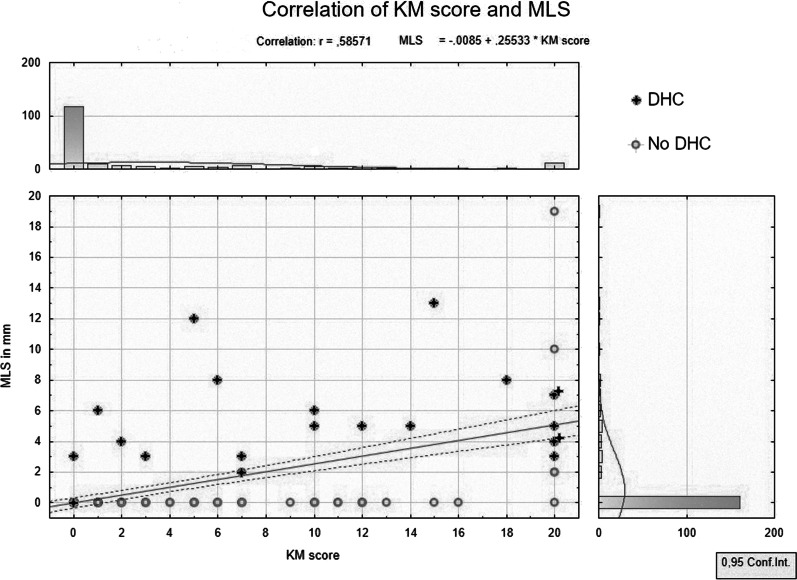
Correlation of KM score and detected midline shift. A rough distribution of measured MLS and calculated KM scores is shown as well as the correlation of them. DHC’s are marked separately. Line—correlation, dotted lines—regression bands (confidence)

For easier handling we simplified the index to a KM Score. If the KM index is lower than 1.005, the KM score is zero. If the KM index is higher than or equal to 1.195, the KM score is 20. Otherwise KM score consists of the rounded 2 decimal places after comma, e.g. KM index 1.14 is KM score 14. As shown in Fig. 5, there is a statistically significant correlation of KM score and MLS as well.

### Risk classification

Based on our retrospective cohort we divided the patients according to the KM index into risk classes for MLS and DHC, as displayed in Table 6.

**Table 6:** Risk classification

KM index	KM score	Risk class	MLS	DHC	Death*	DD
< 1.03	0–2	Low risk	2/129 (2%)	3/129 (2%)	2/129	0
1.03–1.07	3–7	Moderate risk	5/26 (19%)	5/26 (19%)	1/26	0
1.08–1.13	8–13	High risk	4/15 (27%)	4/15 (27%)	1/16	1
> 1.13	14–20	Severe risk	13/16 (81%)	7/16 (44%)	3/16	1

A possible risk classification based on AUC curves

*Death within 7 days after symptom onset; DD = death after DHC within 7 days after symptom onset

## Discussion

Our study in 186 patients suffering from middle cerebral artery infarction shows that the KM index based on an initial CTP scan is an early prediction marker for MLS with a high sensitivity. If KM index is larger than 1.13, close clinical and CT controls and even an early DHC should be seriously considered. Our results can help to identify patients with a high risk for cerebral herniation. This should enable a better and more effective patient care.

Today, neurological worsening und NECT scans are used for monitoring. Except for infarct volume and neurological state of the patient, no prediction marker for MLS is existent. An accurate prediction of the final infarct volume using CTP was observed by Lev et al. (2001) [6] and Flottmann et al. (2017) [7].

In 2008, Dittrich et al [8] showed the accuracy of CTP for risk prediction of malignant brain infarction. An increased infarct permeability [9], and the ratio of ischemic and CSF volume [10] also predicts the development of such a malignant brain infarction.

Keenan et al. determined thresholds for 4 and 8 cm CTP coverage and showed that an infarct volume of greater than 53 ml is being associated with a bad outcome despite rtPA treatment [11].

Davoli et al. [12] analyzed pretreatment predictors, as blood pressure and glucose, and showed that a CTA Alberta Stroke Program Early CT score (ASPECTS) [13] of less than 6 was the best cut-off for malignant infarction in their study.

DESTINY, DESTINY2, DECAP and other studies showed a specific survival benefit for DHC even in elderly patients [1, 2, 14, 15]. In these studies, only neurological worsening and MLS, but not the brain atrophy or the influence of lysis and thrombectomy were analysed. In young patients even small infarcts can lead to MLS and DHC, given smaller subarachnoid spaces and ventricles. Thus, atrophy measures should be also considered as is done by the KM index.

Nevertheless, after recent developments in interventional neuroradiology, no adaption of indication for DHC has been performed. EDEMA-Score [3], which values thrombectomy and lysis together half as important as a high blood glucose level, is highly oscillating and can only be used in the emergency situations. We included all patients with MCA ischemia who received initial CTP imaging, regardless of the size of the occluded vessel, because even the occlusion of a strategic M3 trunk supplying a large brain volume could lead to a midline shift.

In our opinion, the easiest way to assess all of the complex and individual factors (atrophy, thrombectomy result, onset time) is to relate the volumes of brain, penumbra and infarction. We noticed large differences between calculated tissue volumes with reduced CBV and the real infarction volumes, if the thrombectomy was done fast and well [16]. The prediction of infarct and penumbra size as done by KM index, leads to an acceptable approximation of the final infarct volume. We decided to keep the index as simple as possible and as complex as needed despite, results could be better, if we would add additional criteria, e.g. count of thrombectomy maneuvers [17] or collateral blood vessels [18–20]. Although a weak correlation between the KM index and the size of the occluded vessels exists, large embolic shower or an occlusion of two vessels cannot be depicted by the vessel type only, but by the KM index.

## Limitations

A low specificity is acceptable as KM index functions as a screening test with essentially high sensitivity.

Temporal lobe infarction also plays an important role [21], and thus should be taken into account for diagnosis and therapy [22]. Some authors consider lateral displacement of the supratentorial brain to be the main factor and herniation a morphological consequence [23]. Therefore, only the midline shift was analyzed and possible secondary consequences, such as transtentorial herniation or midbrain compression, were not evaluated in our study.

Since our stroke protocol favors a one stop therapy for acute infarction [24] in drip and ship patients, we have to mention a possible selection bias. Not every stroke patient underwent a CTP.

Because of rapid neurological worsening, two patients even underwent a DHC without directly preceding NECT scan, so that a possible MLS could not be detected. These cases were also included in our study for completeness of the cohort. Follow-up scans were performed in individual intervals and in case of neurological worsening.

CTP volumes present only a section of the brain. Hence, the resulting volume ratio are fuzzy and may contain deviations.

As already mentioned, the quality of the CTP plays an important role as well. If motion correction and the selection of brain tissue by the used software doesn’t work well, a calculation of the KM index is imprecise.

The calculated parameters, e.g. the “2.8” weighting factor of the infarct core, strongly depend on the software used. Correlation of infarct volume in CTP and final infarct volume in mTICI 2c and 3 was shown by Austein et al. [25]. “Syngo.via” by Siemens is known for underestimating this volume [25]. Thus, with other software packages this weighting factor might be lower than 2.8.

For easier and faster calculation of the KM index, we provide a simple script at http://www.kmindex.org. Since we always use standard parameters, it should be possible to reproduce our results with the Siemens CT scanner and Syngo.via without any problems. If other systems are used, the parameters may need to be adjusted.

Additional prospective studies for further validation of the KM index and its correlation with clinical outcomes are necessary.

KM index could also be adapted to MRI diagnostics and may be an even better outcome indicator. If CTP or MRI is better has been matter of debate since a long time. In 2015 Liebeskind et al. pleaded for the usage of CTP in acute stroke therapy, while Gonzalez and Lev favored diffusion-weighted MR imaging [26].

### Frequent sources of errors

Sometimes Syngo.via includes bone edges in the MIP in low quality CTP’s, e.g. caused by a skew symmetry plane or by frontal hyperostosis. This can result in an overestimation of the brain volume. Both temporal lobes should be scanned parallel, otherwise MTT-artefacts are observed. Motion correction sometimes can’t handle fast body movements. A badly timed injection of contrast medium can affect the quality of CTP and the results of the KM index as well.

## Conclusion

The decision to perform a DHC requires consideration of multiple factors. One of the most important radiological factors is the MLS, which can be well predicted by the KM index. A KM index > 1.02 shows a moderate risk, and > 1.07 a high risk of MLS. If KM index > 1.13 close clinic and radiographic controls are necessary. Even an early DHC should be seriously considered, if the other clinical factors are consistent with it. But, the decision for DHC keeps a clinical one, mainly based on neurological worsening, patient’s will, prognosis and age.

## Data Availability

The datasets used and analyzed during the current study are available from the corresponding author on reasonable request. Individual data from patients were not published so that no identification can take place.

## Abbreviations

CBF: Cerebral blood flow
CBV: Cerebral blood volume
CCA: Common carotid artery
CFS: Estimated ratio of cerebral fluid space/intracranial volume
CTA: Computed tomography angiography
CTP: Computed tomography perfusion
DESTINY: Decompressive surgery for the treatment of malignant infarction of the middle cerebral artery
DHC: Decompressive hemicraniectomy
EDEMA: Enhanced detection of edema in malignant anterior circulation stroke
I: Estimated ratio of volume of infarct core/intracranial volume
ICA: Internal carotid artery
KM: Kinematics of media ischemia
KMT: Kinematics of media ischemia and time
m: Weighting factor “infarct” using thrombectomy/mTICI
MCA: Middle cerebral artery
MLS: Midline shift
MRI: Magnetic resonance imaging
MIP: Maximum intensity projection
mTICI: Modified thrombolysis in cerebral infarction
MTT: Mean transit time
n: Weighting factor “penumbra” using thrombectomy/mTICI
NECT: Non-enhanced computed tomography
NIHSS: National Institutes of Health Stroke Scale
NOAC: Novel oral anticoagulants
OS: Estimated ratio of occupied intracranial space/intracranial volume
P: Estimated ratio of volume of penumbra/intracranial volume
rtPa: Recombinant tissue plasminogen activator
t: Time exponent

## References

[1] Jüttler E, Schwab S, Schmiedek P, Unterberg A, Hennerici M, Woitzik J (2007). Decompressive surgery for the treatment of malignant infarction of the middle cerebral artery (DESTINY): a randomized controlled trial. Stroke.

[2] Jüttler E, Bösel J, Amiri H, Schiller P, Limprecht R, Hacke W (2011). DESTINY II: decompressive surgery for the treatment of malignant infarction of the middle cerebral artery II. Int J Stroke.

[3] Ong CJ, Gluckstein J, Laurido-Soto O, Yan Y, Dhar R, Lee J-M (2017). Enhanced detection of edema in malignant anterior circulation stroke (EDEMA) score: a risk prediction tool. Stroke.

[4] Almekhlafi MA, Mishra S, Desai JA, Nambiar V, Volny O, Goel A (2014). Not all “successful” angiographic reperfusion patients are an equal validation of a modified TICI scoring system. Interv Neuroradiol.

[5] Maas AI, Stocchetti N, Bullock R (2008). Moderate and severe traumatic brain injury in adults. Lancet Neurol.

[6] Lev MH, Segal AZ, Farkas J, Hossain ST, Putman C, Hunter GJ (2001). Utility of perfusion-weighted CT imaging in acute middle cerebral artery stroke treated with intra-arterial thrombolysis: prediction of final infarct volume and clinical outcome. Stroke.

[7] Flottmann F, Broocks G, Faizy TD, Ernst M, Forkert ND, Grosser M (2017). CT-perfusion stroke imaging: a threshold free probabilistic approach to predict infarct volume compared to traditional ischemic thresholds. Sci Rep.

[8] Dittrich R, Kloska SP, Fischer T, Nam E, Ritter MA, Seidensticker P (2008). Accuracy of perfusion-CT inpredicting malignant middle cerebral artery brain infarction. J Neurol.

[9] Bektas H, Wu T-C, Kasam M, Harun N, Sitton CW, Grotta JC (2010). Increased blood–brain barrier permeability on perfusion CT might predict malignant middle cerebral artery infarction. Stroke.

[10] Minnerup J, Wersching H, Ringelstein EB, Heindel W, Niederstadt T, Schilling M (2011). Prediction of malignant middle cerebral artery infarction using computed tomography-based intracranial volume reserve measurements. Stroke.

[11] Keenan KJ, Christensen S, Inoue M, Mlynash M, Albers GW, Smith WS (2020). Validation and iteration of CT perfusion defined malignant profile thresholds for acute ischemic stroke. Int J Stroke Off J Int Stroke Soc.

[12] Davoli A, Motta C, Koch G, Diomedi M, Napolitano S, Giordano A (2018). Pretreatment predictors of malignant evolution in patients with ischemic stroke undergoing mechanical thrombectomy. J NeuroInterv Surg.

[13] Barber PA, Demchuk AM, Zhang J, Buchan AM (2000). Validity and reliability of a quantitative computed tomography score in predicting outcome of hyperacute stroke before thrombolytic therapy. Lancet.

[14] Rahmig J, Wöpking S, Jüttler E, Uhlmann L, Limprecht R, Barlinn J (2019). Decompressive hemicraniectomy in elderly patients with space-occupying infarction (DECAP): a prospective observational study. Neurocrit Care.

[15] Suyama K, Horie N, Hayashi K, Nagata I (2014). Nationwide survey of decompressive hemicraniectomy for malignant middle cerebral artery infarction in Japan. World Neurosurg.

[16] Lum C, Ahmed ME, Patro S, Thornhill R, Hogan M, Iancu D (2014). Computed tomographic angiography and cerebral blood volume can predict final infarct volume and outcome after recanalization. Stroke.

[17] Seker F, Pfaff J, Wolf M, Ringleb PA, Nagel S, Schönenberger S (2017). Correlation of thrombectomy maneuver count with recanalization success and clinical outcome in patients with ischemic stroke. Am J Neuroradiol.

[18] Liebeskind DS (2005). Collaterals in acute stroke: beyond the clot. Neuroimaging Clin N Am.

[19] de Havenon A, Haynor DR, Tirschwell DL, Majersik JJ, Smith G, Cohen W (2017). Association of collateral blood vessels detected by arterial spin labeling magnetic resonance imaging with neurological outcome after ischemic stroke. JAMA Neurol.

[20] Fisher M, Bastan B (2012). Identifying and utilizing the ischemic penumbra. Neurology.

[21] Barber PA, Demchuk AM, Zhang J, Kasner SE, Hill MD, Berrouschot J (2003). Computed tomographic parameters predicting fatal outcome in large middle cerebral artery infarction. Cerebrovasc Dis.

[22] Treadwell SD, Thanvi B (2010). Malignant middle cerebral artery (MCA) infarction: pathophysiology, diagnosis and management. Postgrad Med J.

[23] Fisher CM (1995). Brain herniation: a revision of classical concepts. Can J Neurol Sci J Can Sci Neurol.

[24] Psychogios M-N, Behme D, Schregel K, Tsogkas I, Maier IL, Leyhe JR (2017). One-stop management of acute stroke patients: minimizing door-to-reperfusion times. Stroke.

[25] Austein F, Riedel C, Kerby T, Meyne J, Binder A, Lindner T (2016). Comparison of perfusion CT software to predict the final infarct volume after thrombectomy. Stroke.

[26] Liebeskind DS, Parsons MW, Wintermark M, Selim M, Molina CA, Lev MH (2015). Computed tomography perfusion in acute ischemic stroke: is it ready for prime time?. Stroke.

[27] Khadhraoui E, Müller SJ, Riedel CH (2020). 55. Jahrestagung der Deutschen Gesellschaft für Neuroradiologie e.V. Clin Neuroradiol.

